# 2208: Patient satisfaction with the Michigan Surgical and Health Optimization Program (MSHOP): A mixed methods study

**DOI:** 10.1017/cts.2017.146

**Published:** 2018-05-10

**Authors:** Martin Gruca, Angela K. Lyden, Anjana M. Kumar, Elizabeth A. Jackson

## Abstract

OBJECTIVES/SPECIFIC AIMS: This project has 2 overarching objectives: (1) to investigate the acceptability of the Michigan Surgical and Health Optimization Program (MSHOP) among referred patients, and to describe individual motivations behind enrollment Versus nonenrollment; and (2) to identify patient and program related factors associated with adherence and LOS and readmission rates. METHODS/STUDY POPULATION: Hypothesis—(1) MSHOP participants will report overall satisfaction with the program. Individuals that are satisfied with the program will be likely to perceive the program as effective. Subjects that declined MSHOP will be more likely to perceive their outcomes as immutable. (2) MSHOP patients will have shorter hospital stays and fewer readmission compared with patients who declined MSHOP. Methods—this study will use both qualitative and quantitative methods to investigate patient experiences and program efficacy. First, a convenience sample of patients who were referred to the MSHOP within the previous 12 months will participate in structured interviews to assess program acceptability, patient satisfaction with individual components of MSHOP, and perception of program efficacy. Interviews will also include patients who declined to enroll in MSHOP. Interviews for these subjects will include questions that assess why patients chose to decline enrollment. Second, there will be a retrospective cohort study comparing hospital outcomes among patients who enrolled in MSHOP Versus those who chose not to enroll. Analysis—interviews will be recorded and transcribed for thematic analysis to identify patterns associated with satisfaction or dissatisfaction with the MSHOP. Multivariate regression will be used to determine effect of MSHOP participation on postsurgical length-of-stay and 30-day readmission rate. Demographics and procedure type will be included as covariates. RESULTS/ANTICIPATED RESULTS: In total, 28 interviews have been transcribed, and are in the initial stages of thematic analysis. Interviews have thus far suggested that patients have been satisfied with MSHOP and would recommend the intervention to other patients. Retrospective data regarding hospital length of stay for MSHOP patients from September 2014 to December 2016 has been acquired and is being processed. The characteristics of patients that tend to participate more actively in MSHOP will be explored. We anticipate that active participation in the MSHOP will be associated with shorter hospital stays and fewer readmissions. DISCUSSION/SIGNIFICANCE OF IMPACT: This study will be one of the first to characterize patient perception of MSHOP, in particular its use of tracking step counts and breathing exercises to promote a form of prehabilitation that is easier to integrate into daily life. This project will investigate MSHOP’s effect on patient outcomes, as well as explore factors that may associate with better patient adherence and outcomes. This would help further optimize the MSHOP as an intervention.

